# A universal artificial diet for corn rootworm (*Diabrotica* spp.) larval biopesticide assays

**DOI:** 10.3389/finsc.2024.1392198

**Published:** 2024-07-02

**Authors:** Khanh-Van Ho, Bruce E. Hibbard, Thu T. H. Do, Adrian J. Pekarcik, Man P. Huynh

**Affiliations:** ^1^ Department of Chemistry, University of Missouri, Columbia, MO, United States; ^2^ Molecular Imaging and Theranostics Center, University of Missouri, Columbia, MO, United States; ^3^ Department of Post-harvest Technology, Can Tho University, Can Tho, Vietnam; ^4^ Plant Genetics Research Unit, USDA-Agricultural Research Service, Columbia, MO, United States; ^5^ Division of Plant Science & Technology, University of Missouri, Columbia, MO, United States; ^6^ North Central Agricultural Research Laboratory, USDA- Agricultural Research Service, Brookings, SD, United States; ^7^ Department of Plant Protection, Can Tho University, Can Tho, Vietnam

**Keywords:** western corn rootworm, northern corn rootworm, southern corn rootworm, insect rearing, artificial diet, diet assay

## Abstract

We identified a single diet formulation that can be used for three *Diabrotica* species including southern (SCR), western (WCR), and northern corn rootworm (NCR) by evaluating the performance of these pests on specialized diets (F9800B diet for SCR, WCRMO-2 diet for WCR, and NCRMO-1 diet for NCR) and a larval diet (F9772 diet) widely used for lepidopteran species. After 10 days of rearing, the WCRMO-2 diet yielded better or equal larval growth and development of all three rootworm species compared to other diets. For SCR larvae, the WCRMO-2 diet outperformed other diets. Larval fresh weight, percent molt to 2nd instar, and percent molt to 3rd instar on the WCRMO-2 diet were 12-fold, 2.7-fold, and 14-fold increases, respectively compared to that of the F9800B diet. Significantly more SCR larvae survived on the WCRMO-2 diet (98.9%) than on the F9800B diet (90.6%). The WCRMO-2 diet supported WCR and NCR larvae equal to the NCRMO-1 diet and better than other diets. The F9772 diet was the worst diet of all examined species. The availability of a universal diet (the WCRMO-2 diet) for the three *Diabrotica* species would facilitate research programs to monitor resistance development and develop new control tactics targeting these important pests.

## Introduction

Three *Diabrotica* species including the western corn rootworm (WCR, *Diabrotica virgifera virgifera* LeConte), the northern corn rootworm (NCR, *Diabrotica barberi* Smith & Lawrence), and the southern corn rootworm (SCR, *Diabrotica undecimpunctata howardi* Barber) are the most important insect pests of maize (*Zea mays* L.) in the United States (1, 2), causing approximately $2 billion each year in yield losses and control costs to U.S. maize growers (3). Most of the damage caused by the corn rootworm complex primarily arises from larval feeding on maize roots (4), while adults at extremely high densities can cause yield losses via feeding on green silks of maize plants (5). Management of the corn rootworm species has continuously presented challenges to maize growers since WCR and NCR are highly adaptive insect pests that have evolved resistance to nearly every control tactic targeting them including crop rotation, chemical insecticides, and transgenic maize hybrids expressing insecticidal crystalline toxins from *Bacillus thuringiensis* (Bt) Berliner (6–13).

The U.S. Environmental Protection Agency (EPA) has mandated resistance monitoring programs to mitigate the resistance development of insect pests of Bt maize (14). One component of these programs is to determine changes in the susceptibility of insect populations in regions of high adoption of transgenic maize over time. Diet assays, whereby insecticidal toxins are typically overlaid onto an artificial diet for evaluating their efficacy on insects, and on-plant assays allow determining the susceptibility of corn rootworms to target compounds and entomopathogenic agents. These tools are crucial for the resistance monitoring programs as well as research discovery programs of new insecticidal toxins and other control agents (e.g., entomopathogenic nematodes) to manage the insect pests of maize (15–22).

Artificial diets have been developed specifically for each of the three major *Diabrotica* species. The diet development began with SCR since this species has no diapause phase and a short lifecycle (23, 24). An attempt to develop a diet for rearing SCR occurred in 1971 (24). This agar-based diet consisted of wheat germ, casein, sucrose, linseed oil, and cellulose as macronutrient ingredients, cholesterol, vitamin and salt mixtures for micronutrients, a number of diet preservatives (e.g., formalin, methyl paraben), agar, and water. The latest publicly available diet for SCR larvae was introduced in 1985 based on modifications in the proportions of the ingredients in the first SCR diet (25). Later, a version of this diet free of formalin was developed and commercially available (F9800B, Frontier Agricultural Sciences, Newark, DE). Contamination (e.g., bacteria, fungi) has been a major issue in rearing SCR and WCR larvae on artificial diets (25, 26). Formalin was initially included in the SCR diet to reduce contamination. This preservative agent is no longer used in the diets for corn rootworms since contamination was effectively prevented via applications of egg sterilization and clean laboratory practices (27–29). The inclusion of formalin in the diet formulations also greatly reduced larval survival and weight of all three rootworm species including SCR, WCR, and NCR (25, 29, 30).

An initial diet for WCR larvae was developed in 2002 (29). This diet formulation was a modification of ingredient amounts in the SCR diet plus an addition of maize root powder and an exclusion of formalin (29). Proprietary diet formulations of WCR larvae have been developed by each of the major maize seed companies that were likely happened since that time (27). Later, an improved WCR diet was developed based on an optimization of the ingredients in the initial diet using response surface modeling combined with mixture designs (28). This diet was compatible with each of all four current Bt proteins used for WCR control (31). However, the diet formulations for WCR larvae contained maize root powder which is not available for purchase (28, 29). The equipment required to produce the maize root powder is prohibitively expensive, thus limiting the practical use of diets consisting of this ingredient (32). Recently, a new WCR formulation has been developed that excluded the maize root powder, comprised of available ingredients, and is commercially available (WCRMO-2, Frontier Agricultural Sciences) (33). This WCR larval diet was developed by systematically exploring eight protein ingredients from animal, plant, and yeast sources in order to eliminate the maize root powder. The new formulation supported the performance of WCR larvae equal to or better than all previous formulations including publicly available and proprietary formulations (33). More recently, a larval diet specialized for NCR was created using a multidimensional approach (30). The NCR diet had the same ingredients used in the WCRMO-2 diet plus an addition of casein. This formulation has been used to determine the baseline susceptibility of several NCR populations to all four Bt proteins and transgenic maize targeting corn rootworms (18, 34).

A single artificial diet that can be used for rearing all three *Diabrotica* spp. would be highly advantageous to facilitate standardized corn rootworm resistance monitoring assays and accelerate discovery efforts related to new insecticides and their related products to control these pests. Evaluating rootworm toxins on differing diets does not allow direct comparisons of different toxins or their efficacy on different corn rootworm species because of differences between artificial diet formulations. In fact, nutrition may significantly affect the susceptibility of corn rootworm larvae to Bt toxins as has been shown for lepidopteran insects (35, 36). The cabbage looper larvae (*Trichoplusia ni* Hübner) were more susceptible to a Cry1Ac toxin when reared on a diet with a low carbohydrate: protein ratio (10:90) than a diet with a high carbohydrate: protein (65:35) ratio (36). Our goal of the present study was to identify an artificial diet that can be used for all three corn rootworm species (SCR, WCR, and NCR) by evaluating the performance of these species on their specialized diets (F9800B diet for SCR, WCRMO-2 diet for WCR, NCRMO-1 diet for NCR) plus F9772 diet that is widely used for rearing various lepidopteran species. The evaluation was based on life history parameters of three corn rootworm larvae (weight, molt, and survival) reared on these diets.

## Materials and methods

### Artificial diets

The F9800B, WCRMO-2, NCRMO-1, and F9772 diets were purchased from Frontier Agricultural Sciences. The F9800B diet had the same ingredients as the latest publicly available diet for SCR larvae (25), except for the exclusion of formalin in the F9800B diet. Formulations of the WCRMO-2 and NCRMO-1 diets were published previously (30, 33), while information on the F9772 formulation is available at Frontier Agricultural Sciences (insectrearing.com/product/general-purpose-lepidoptera). The NCRMO-1 diet is not commercially available and was prepared by Frontier Agricultural Sciences based on its publicly available formulation (30).

### Insects and egg treatments

Non-diapausing eggs of a WCR colony were obtained from the USDA-ARS Plant Genetics Research Unit in Columbia, MO. This WCR colony was developed from non-diapausing eggs originally purchased from Crop Characteristics (Farmington, MN) (37). Non-diapausing eggs of an NCR colony were supplied by the USDA-ARS North Central Agricultural Research Laboratory in Brooking, SD. The NCR colony was developed from a diapausing colony via selective breeding for early egg hatched as described previously (38). Non-diapausing eggs of SCR (Crop Characteristics) were obtained from a non-diapausing colony that has >100 generations maintained on corn (39).

The eggs of all three insect species were provided in Petri dishes with 70-mesh sieved soil. Upon arrival, egg plates were incubated at 25°C in complete darkness in an incubator (Percival, Perry, IA) until emerging larvae were seen. After ~10% of the eggs were hatched, the eggs were surface-sterilized as described previously (27). Briefly, the eggs were washed from soil by rinsing the mixtures of eggs and soil with tap water in a 60-mesh sieve (Hogentogler & Co. Inc., Columbia, MD). The eggs free of soil retained in the 60-mesh sieve were collected into a 50 mL beaker. Subsequently, these eggs were treated with 10 ml of undiluted Lysol (Clean & Fresh Multi-Surface Cleaner, Reckitt Benckiser, Parsippany, NJ) for 3 min and then triple rinsed with purified water after the undiluted Lysol was removed. The treated eggs were exposed to 10 ml of 10% formalin (HT501128, Sigma Aldrich, St. Louis, MO), followed by another triple rinse with purified water after removing the formalin solution. Lastly, the eggs were dispensed to a coffee filter paper (Pure Brew, Rockline Industries, Sheboygan, WI) placed inside a 16-oz container with a lid (LG8RB-0090 & DM16R-0090, Solo Cup Company, Lake Forest, IL). The egg container was included at 25°C overnight in darkness. Neonate larvae (< 24 h old) were used for the insect diet bioassays.

### Diet preparation

The F9800B and F9772 diets were prepared according to the manufacturer’s procedure, while the WCRMO-2 and NCRMO-1 diets were poured as described previously (30, 40). Briefly, agar was added to purified water and the agar solution was boiled using a microwave (Hamilton Beach, Glen Allen, VA) until the agar was completely melted. Next, dry mixtures of the F9800B or F9772 diets were immediately added to the agar solution, whereas dry mixtures of the WCRMO-2 or NCRMO-1 diets were added to the agar solution when it cooled to 65°C. The temperatures of the WCRMO-2 and NCRMO-1 diets were measured using an infrared thermometer (IR002, Ryobi, Fuchu, Hiroshima, Japan). Subsequently, KOH solution was added to the diet mixtures of the F9800B, WCRMO-2, and NCRMO-1 diets. The diet mixtures were blended for 30 seconds to mix thoroughly using a stir-bar on a stirring hot plate held at 65°C (CimarecTM, Thermo Scientific). Finally, the diet solutions were dispensed into wells (200 µl per well) of a 96-well plate (3370, Corning Inc., Corning, NY) using a repeater pipette (HandyStep^®^ S, Brandtech, Essex, CT). The diet plates were stored in a refrigerator at 4°C and used for assays within 2 days.

### Insect diet bioassays

The insect diet bioassays were conducted inside a biological cabinet (SterilGARD^®^ III Advance cabinet, Sanford, ME) as described previously (28). All materials used were surface-sterilized using UV light for 5 min prior to the assays. When making experimental diet plates as described above, each diet formulation (WCRMO-2, NCRMO-1, F9800B, or F9772 diet) was randomly assigned to a 12-well row of the 96-well plate and replicated 8 times in different diet plates. One neonate larva of each insect species was transferred to each well using a fine paintbrush. Each replicate included 12 larvae that fed on designed diets in 12 wells/row of the 96-well plate. After the larval infestation, the plates were sealed using a sealing film (TSS-RTQ-100, Excel Scientific, Inc., Victorville, CA). An insect pin (#0) was used to make a hole over each well in the sealing cover for ventilation. These plates were kept in an incubator (Percival, Perry, IA) at 25°C in darkness for 10 days. The assays were run in three cohorts at different times. Each cohort that included all three corn rootworm species was performed at the same time. Each cohort had 8 replicates per diet treatment. There were 24 replicates per diet treatment in total.

The performance of all three corn rootworm species on different diets was evaluated by life history parameters (survival, weight, and molt) of these larvae reared on these diets for 10 days. The diet assays for corn rootworm (*Diabrotica* spp.) larvae typically run up to 10 days (15, 20, 22). Larval molting was recorded daily during the assays, while larval fresh weight and survival were determined at the end of the assays. To collect larval fresh weight, all live larvae were identified and then the experimental plates were frozen in a freezer overnight. Subsequently, the plates were thawed for 30 min and the larvae that survived in each treatment were then pooled per replicate (12 possible) and weighed using a micro balance (MSU 6.6S-000-DM, Sartorius Lab Instruments GmbH & Co. KG Göttingen, Germany).

### Data analysis

Survival and molting data were determined by dividing the number of live larvae and successful larval molt from 1st to 2nd instar and from 1st to 3rd instar per replicate, respectively, by the initial number of larvae infested and multiplying by 100 to obtain percentages. Weight per larva (mg) was calculated by dividing the fresh weight by the number of larvae that were alive per replicate. If all larvae per replicate were dead, larval weight for this replicate was recorded as 0.

The experiment was designed as a 2-factor factorial design (diet × insect species). The life history data (percent survival, weight, percent molt to 2nd instar, and percent molt to 3rd instar) were analyzed with analysis of variance (ANOVA) in generalized linear mixed models (PROC GLIMMIX) in SAS 9.4 (SAS Institute, Cary, NC). Diet and insect species were the fixed effects and replication was the random variable. Differences between treatments were determined using Fisher’s least significant difference (LSD) at p < 0.05. The percent variables (survival and molt) were arcsine square-root transformed and the numeric variable (weight) was square-root transformed prior to the analysis to meet assumption of normality and homoscedasticity. The untransformed data were presented as MEAN ± SEM.

## Results

**Figure 1:**
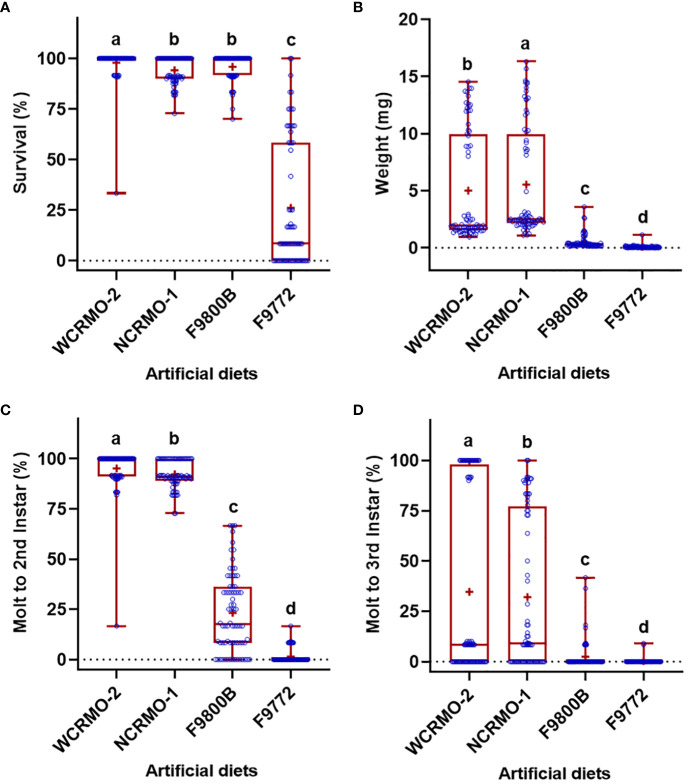
Percent survival **(A)**, fresh weight **(B)**, percent successful completion of molt to 2nd instar **(C)** and 3rd instar **(D)** for three *Diabrotica* species (SCR, southern corn rootworm; WCR, western corn rootworm; and NCR, northern corn rootworm) reared on four different diets for 10 days. Box plots with median (red line), the 25th and 75th percentiles (bottom and top of box, respectively), the 5th and 95th percentiles (whiskers), and means (+) are shown. Open dots are data points. Boxes with different letters are significantly different (*P* < 0.05). Untransformed data were presented, while analyses were performed with square-root transformed or arcsine square-root transformed data.

**Figure 2:**
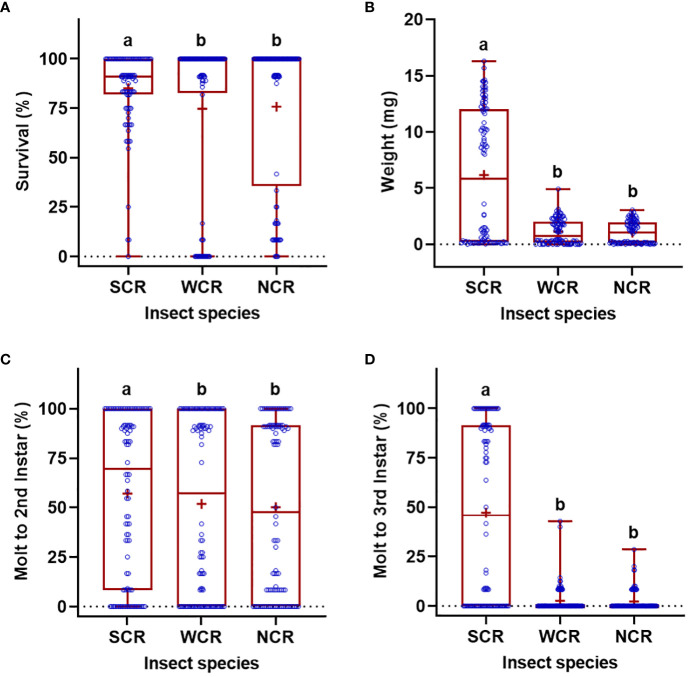
Percent survival **(A)**, fresh weight **(B)**, percent successful completion of molt to 2nd instar **(C)** and 3rd instar **(D)** for three *Diabrotica* species (SCR, southern corn rootworm; WCR, western corn rootworm; and NCR, northern corn rootworm) reared on four different diets for 10 days. Box plots with median (red line), the 25th and 75th percentiles (bottom and top of box, respectively), the 5th and 95th percentiles (whiskers), and means (+) are shown. Open dots are data points. Boxes with different letters are significantly different (*P* < 0.05). Untransformed data were presented, while analyses were performed with square-root transformed or arcsine square-root transformed data.

Significant differences in the life history parameters (weight, survival, and molting) were found among diets, insect species, and their interactions (**Table 1**). With respect to the diets, when data of all rootworm species were combined, the WCRMO-2 diet generally supported better larval growth and development than other diets evaluated, followed by NCRMO-1, F9800B, and F9772 diets (**Figure 1**). Rootworm neonates reared on the WCRMO-2 diet had the highest percent survival, percent molt to 2nd, and percent molt to 3rd instar. The NCRMO-1 diet had the highest larval weight and significantly higher percent molt to 2nd instar and molt to 3rd instar than the F9800B and F9772 diets. There was no significant difference in percent survival between the F9800B and NCRMO-1 diets. Significantly fewer larvae survived and molted when reared on the F9772 diet than other diets (**Figure 1**). Among the three corn rootworm species, SCR larvae had the highest percent survival (85.1%), weight (6.2 mg), percent molt to 2nd instar (57.0%), and percent molt to 3rd instar (47.1%), whereas no significant differences in these life history parameters were observed on WCR and NCR larvae when data of all diets were combined (**Figure 2**).

**Table 1:** The performance of three corn rootworm species (SCR: southern corn rootworm, WCR: western corn rootworm, and NCR: northern corn rootworm) reared on four diets including WCRMO-2, NCRMO-1, F9800B, and F9772 diets.

Analysis	Effect	df	F value	P value
Survival	Diet	3, 253	506.27	<0.0001
	Insect	3, 253	8.09	0.0004
	Insect × Diet	6, 253	37.07	<0.0001
Weight	Diet	3, 253	1443.76	<0.0001
	Insect	3, 253	913.14	<0.0001
	Insect × Diet	6, 253	166.10	<0.0001
Molt to 2nd instar	Diet	3, 253	922.32	<0.0001
	Insect	3, 253	7.88	0.0005
	Insect × Diet	6, 253	5.85	<0.0001
Molt to 3rd instar	Diet	3, 253	308.07	<0.0001
	Insect	3, 253	669.44	<0.0001
	Insect × Diet	6, 253	174.73	<0.0001

**Figure 3:**
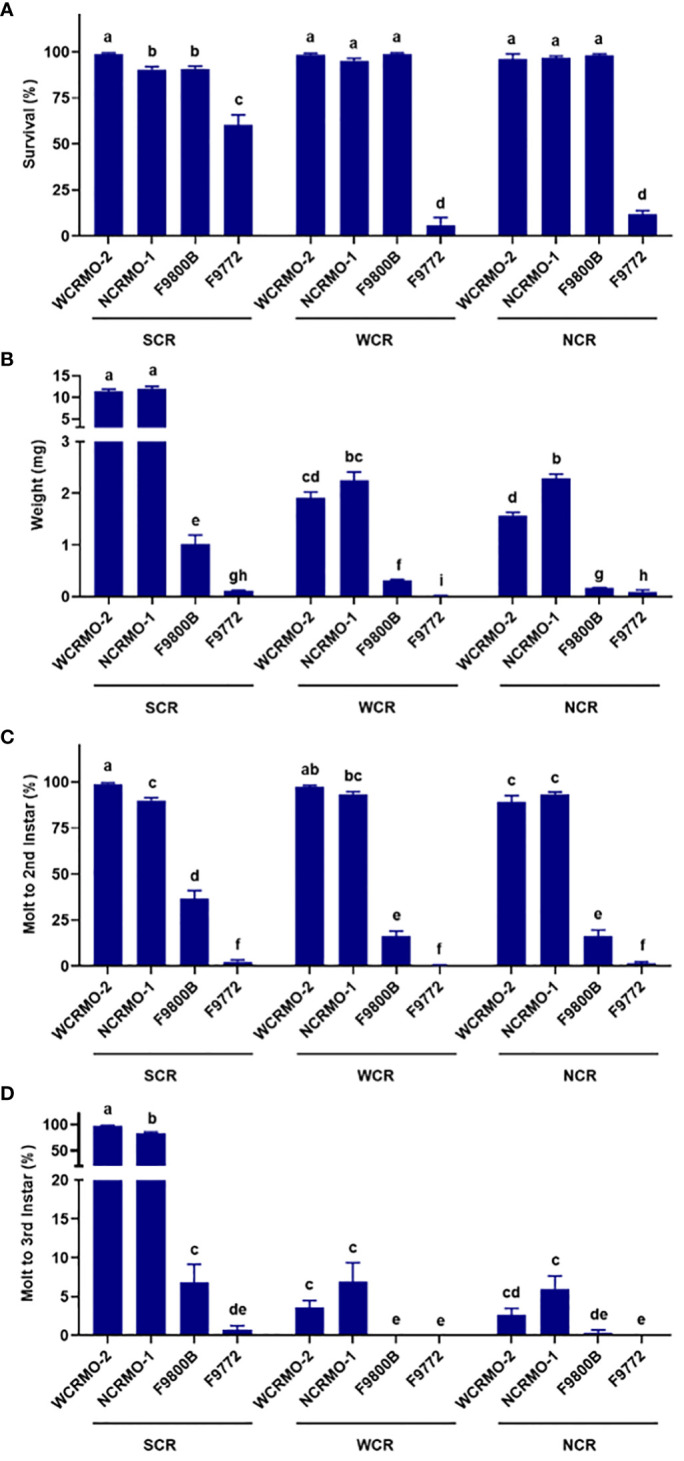
Percent survival **(A)**, fresh weight **(B)**, percent successful completion of molt to 2nd instar **(C)** and 3rd instar **(D)** for three *Diabrotica* species (SCR, southern corn rootworm; WCR, western corn rootworm; and NCR, northern corn rootworm) reared on four different diets for 10 days. Bars with different letters are significantly different (P < 0.05). Untransformed data were presented, while analyses were performed with square-root transformed or arcsine square-root transformed data. Mean ± SEM.

Southern corn rootworm. The WCRMO-2 diet had significantly higher percent survival (98.9%), molt to 2nd instar (98.9%), and molt to 3rd instar (97.8%) than other diets (**Figure 3**). Average fresh weight of SCR larvae on the WCRMO-2 and NCRMO-1 diets were 11.5 mg and 12.0 mg, while average fresh weight on the F9800B and F9772 diets were only 1.0 mg and 0.11 mg, approximately 11.5- and 105-fold differences, respectively, compared to the WCRMO-2 diet. No significant differences in percent survival between the NCRMO-1 (90.5%) and F9800B (90.6%) diets, while only 60.5% SCR larvae survived on the F9772 diet. Significantly more larvae molted to 2nd instar (90.1%) and 3rd instar (83.2%) on the NCRMO-1 diet than the F9800B (36.6% and 6.9%) and F9772 (2.5% and 0.7%) diets (**Figure 3**).

Western corn rootworm. Both the WCRMO-2 and NCRMO-1 diets supported WCR larval growth and development better than the F9800B and F9772 diets (**Figure 3**). No significant difference between the WCRMO-2 and NCRMO-1 diets on all life history parameters measured (survival, weight, and molt) was observed. Larval survivorship was over 95% on the F9800B, WCRMO-2, and NCRMO-1 diets, while only a few larvae survived on the F9772 diet (5.9%). The fresh weight of WCR larvae on the F9800B and F9772 diets were 0.32 mg and 0.01 mg, respectively, which were significantly smaller than that for the WCRMO-2 (1.9 mg) and NCRMO-1 (2.3 mg) diets. Significantly fewer larvae molted to 2nd instar when reared on the F9800B diet (16.4%) than the WCRMO-2 (97.3%) and NCRMO-1 (93.3%) diets, whereas less than 1% larvae molted when fed on the F9772 diet. Less than 7% larvae molted to 3rd instar when WCR neonates were reared on the WCRMO-2 and NCRMO-1 diets, while no 3rd instar larvae were observed on the F9800B and F9772 diets (**Figure 3**).

Northern corn rootworm. Survivorship of NCR larvae was over 96% on the WCRMO-2, NCRMO-1, and F9800B diets, while significantly lower percent survival was observed on the F9772 diet (11.8%) (**Figure 3**). The NCRMO-1 diet had the highest larval fresh weight (2.3 mg) which was significantly higher than the WCRMO-2 diet (1.6 mg). NCR larvae reared on the F9800B (0.17 mg) and F9772 (0.09 mg) diets were tiny compared to other diets. Significantly more larvae molted to 2nd instar on the WCRMO-2 (89.2%) and NCRMO-1 (93.5%) diets compared to the F9800B (16.5%) and F9772 (1.7%) diets. The WCRMO-2 and NCRMO-1 produced less than 6% 3rd instar larvae, whereas other diets had basically no 3rd instar larvae (**Figure 3**).

## Discussion

Artificial diets are critical to entomological research as they can aid for bioassays of potential insecticides, entomopathogens, and insect-plant resistance characteristics (41). Over the past 50 years since the first diet for *Diabrotica* spp. was developed in 1971, diet formulations have been developed and refined for each of three corn rootworm species including SCR, WCR, and NCR (24, 25, 29, 30, 33). In the present study, by evaluating the performance of these corn rootworm species (SCR, WCR, and NCR) on their specialized diets and a general larval diet of lepidopteran insects, we identified a single formulation (WCRMO-2 diet) that can be used for all three corn rootworm species.

Our results indicated that the WCRMO-2 diet can be used as a universal diet for all three rootworm species. In fact, for rearing SCR larvae, the WCRMO-2 diet outperformed other diets including the NCRMO-1 diet, the F9800B diet, the specialized larval diet for SCR, and the F9772 diet (**Figure 1**). Noticeably, larval fresh weight, percent molt to 2nd instar, and percent molt to 3rd instar of SCR on the WCRMO-2 diet for 10 days were 12-fold, 2.7-fold, and 14-fold increases, respectively compared with that of those on the F9800B diet. Significantly more larvae survived on the WCRMO-2 diet (98.9%) than on the F9800B diet (90.6%). No significant difference in the life history parameters (survival, weight, and molt) of WCR and NCR larvae on the WCRMO-2 and NCRMO-1 diets was observed, whereas these diets yielded significantly better growth and development of WCR and NCR larvae compared to the F9800B diet. The F9772 diet was the worst diet for all three rootworm species. A similar pattern of NCR larval development on the NCRMO-1 and F9800B diets was previously observed (30). The F9800B diet is an inferior diet for NCR larvae that supported significantly lower NCR development than the NCRMO-1 diet (30).

Although larvae of the three rootworm species examined primarily feed on maize roots as the natural host plant (42), it’s noteworthy that WCR and NCR larvae are specialist herbivores feeding exclusively on maize roots and SCR larvae are generalist herbivores that can feed over 100 plant species (43–47). The WCRMO-2 and NCRMO-1 diets are the diets that sufficiently support the growth and development of WCR and NCR larvae (30, 33). With the high specialization of WCR and NCR to maize as their solely natural host plant (48), it’s likely that these diet formulations provide sufficient nutrients as WCR and NCR larvae acquire from maize roots. Consequently, the WCRMO-2 and NCRMO-1 diets produced better performance for SCR larvae than the F9800B diet (**Figure 3**). The F9800B diet is a newer version of the most recent public diet for SCR diet developed by Marrone, Ferri, Mosley and Meinke (25). Larval development of SCR on the Marrone et al. diet lag behind maize roots and this diet required 6 generations of selection for larval vigor to establish a SCR strain that developed on the Marrone et al. diet comparable to maize roots (25). Significantly lower developmental rates (weight and molt) of WCR and NCR larvae on the F9800B diet than the WCRMO-2 and NCRMO-1 diets were also observed (**Figure 3**).

Our results revealed similarities and differences in the growth and development of the corn rootworm species fed on different diets. Among the three rootworm species, SCR had the fastest development and the highest weight, whereas the development of WCR and NCR larvae roughly shared a similar pattern (**Figure 2**). These inherent patterns may arise from the differences in genotype of these species. Interestingly, after 10 days of feeding on the F9772 diet, the survivorship was significantly higher for SCR larvae (60.5%) than WCR (5.9%) and NCR (11.8%) larvae, while the survivorship of all three rootworm species was over 90% on all rootworm diets. The F9772 diet is a general diet for lepidopteran insects and is not optimized for rearing corn rootworm larvae. This diet likely lacks key nutrient factors for corn rootworm development, causing significantly detrimental effects on these insect pests. Further research could focus on the identification of key nutrient factors limiting survival of corn rootworm larvae, which may facilitate the development of control tools for these important pests.

The availability of corn rootworm diets has greatly facilitated research programs for management programs of these pests as well as expansion of their basic biology. The artificial diets for corn rootworm species have been utilized for monitoring resistance development of these pests to Bt maize and research programs to develop new control agents for these insect pests of maize (15–22). This is the first report of a single formulation (WCRMO-2) that can be used for all three important rootworm species. The WCRMO-2 diet supported the growth and development of SCR, WCR, and NCR larvae equal to or better than other diets developed for these pests. This formulation would facilitate standardized corn rootworm resistance monitoring assays and accelerate product discovery. Our ongoing research includes the development of a system for continuous rearing of corn rootworms solely on artificial diets for multiple generations.

## Data availability statement

The raw data supporting the conclusions of this article will be made available by the authors, without undue reservation.

## Ethics statement

The manuscript presents research on animals that do not require ethical approval for their study.

## Author contributions

KH: Formal analysis, Validation, Visualization, Writing – original draft, Writing – review & editing. BH: Conceptualization, Funding acquisition, Resources, Writing – review & editing. TD: Data curation, Writing – review & editing. AP: Resources, Writing – review & editing. MH: Conceptualization, Data curation, Investigation, Methodology, Project administration, Supervision, Validation, Writing – original draft, Writing – review & editing.
